# Chromophoric cerium oxide nanoparticle-loaded sucking disk-type strip sensor for optical measurement of glucose in tear fluid

**DOI:** 10.1186/s40824-023-00469-5

**Published:** 2023-12-18

**Authors:** Sijin Park, Dong Yeon Nam, Hee-Jae Jeon, Jae Hoon Han, Dawon Jang, Juil Hwang, Yeong-Seo Park, Young-Geun Han, Young Bin Choy, Dong Yun Lee

**Affiliations:** 1 Department of Bioengineering, College of Engineering, and BK FOUR Biopharmaceutical Innovation Leader for Education and Research Group, Hanyang University, 222 Wangsimni-Ro Seongdong-Gu, Seoul, 04763 Republic of Korea; 2 College of Engineering, Interdisciplinary Program in Bioengineering, Seoul National University, 1 Gwanak-Ro Gwanak-Gu, Seoul, 08826 Republic of Korea; 3 Department of Mechanical and Biomedical Engineering, Kangwon National University, 1 Gangwondaehak-Gil, Chuncheon, 24341 Republic of Korea; 4 Department of Physics, College of Natural Sciences, Hanyang University, 222 Wangsimni-Ro Seongdong-Gu, Seoul, 04763 Republic of Korea; 5 Department of Biomedical Engineering, Seoul National University College of Medicine, 101 Daehak-Ro Jongno-Gu, Seoul, 03080 Republic of Korea; 6 Institute of Medical & Biological Engineering, Medical Research Center, Seoul National University, 101 Daehak-Ro Jongno-Gu, Seoul, 03080 Republic of Korea; 7 Institute of Nano Science and Technology (INST) and Institute for Bioengineering and Biopharmaceutical Research (IBBR), Hanyang University, 222 Wangsimni-Ro Seongdong-Gu, Seoul, 04763 Republic of Korea; 8 Elixir Pharmatech Inc, 222 Wangsimni-Ro Seongdong-Gu, Seoul, 04763 Republic of Korea

**Keywords:** Sucking disk-type (SD) strip biosensor, Cerium oxide nanoparticles (CNPs), Image processing algorithms, Tear glucose, Diabetes

## Abstract

**Background:**

Noninvasive monitoring of tear glucose levels can be convenient for patients to manage their diabetes mellitus. However, there are issues with monitoring tear glucose levels, such as the invasiveness of some methods, the miniaturization, inaccuracy, or the high cost of wearable devices. To overcome the issues, we newly designed a sucking disk-type (SD) strip biosensor that can quickly suck tear fluid and contains cerium oxide nanoparticle (CNP) that causes a unique color change according to the glucose level of the tear without complicated electronic components.

**Methods:**

The SD strip biosensor composed of three distinct parts (tip, channel, and reaction chamber) was designed to contain the sensing paper, onto which tear fluid can be collected and delivered. The sensing paper treated with CNP/APTS (aminopropyltriethoxysilane) /GOx (glucose oxidase) was characterized. Then we carried out the reliability of the SD strip biosensor in the diabetic rabbit animals. We quantitatively analyzed the color values of the SD strip biosensor through the colorimetric analysis algorithm.

**Results:**

We contacted the inferior palpebral conjunctiva (IPC) of a diabetic rabbit eye using an SD strip biosensor to collect tears without eye irritation and successfully verified the performance and quantitative efficacy of the sensor. An image processing algorithm that can optimize measurement accuracy is developed for accurate color change measurement of SD strip biosensors. The validation tests show a good correlation between glucose concentrations measured in the tear and blood.

**Conclusion:**

Our findings demonstrate that the CNP-embedded SD strip biosensor and the associated image processing can simply monitor tear glucose to manage diabetes mellitus.

**Graphical Abstract:**

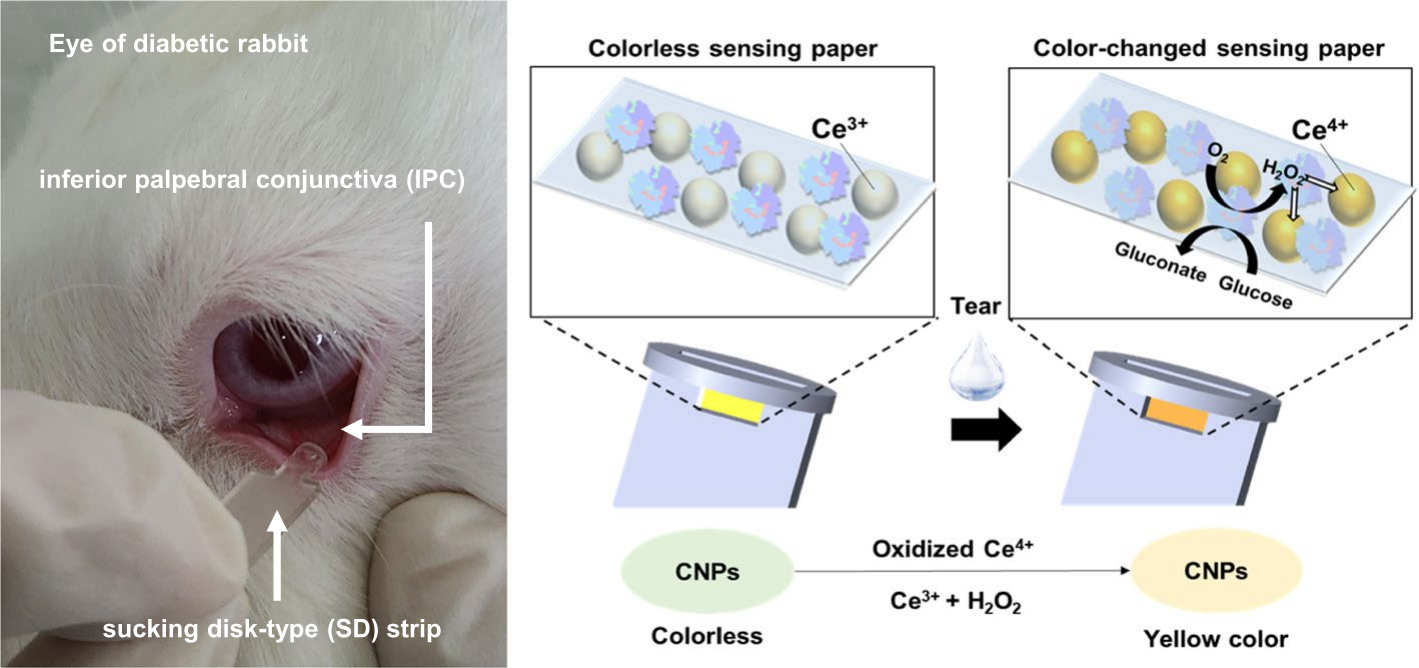

**Supplementary Information:**

The online version contains supplementary material available at 10.1186/s40824-023-00469-5.

## Background

Diabetes is a chronic disease that can lead to serious complications such as cardiovascular diseases, diabetic retinopathy, diabetic nephropathy, and diabetic neuropathy [1, 2]. Early diagnosis and proper blood glucose management are important to control and prevent the progression of the disease and its complications [3–8]. Conventional point-of-care glucose monitoring systems and blood glucose self-testing (BGST) are commonly used in diabetes management [9]. The standard technology for monitoring blood glucose concentration is based on blood collection, which a drop of blood is invasively taken by pricking a finger [10, 11]. The collected blood is drawn on a test strip containing glucose oxidase or glucose dehydrogenase to check the glucose concentration [12, 13]. Because blood glucose diagnosis requires repetitive pricking of a finger, the finger-pricking test is usually inconvenient and can cause pain and stress for the patient, as well as the risk of a blood-borne infection [14, 15]. As a result, patient compliance with frequent and routine examinations is often low, increasing the risk of complications [16, 17].

Recently, many studies have been conducted on methods for non-invasively measuring blood glucose using body fluids such as sweat, saliva, urine, and tears [18–21]. Measuring glucose levels in sweat, saliva, or urine is not always accurate and has the potential for toxicity as well as interference from other substances [22–24]. In contrast, tear fluid has been identified as a less interfering non-invasive source for monitoring glucose levels in diabetes patients [25]. Compared to conventional finger-pricking tests, monitoring tear glucose levels provides a less invasive and continuous monitoring option [26, 27]. To this end, various measurement methods using electric sensors or optical arrangements have been proposed and investigated [27–34]. While continuous monitoring of tear fluids using electronic glucose sensors is a prevalent method, it's noteworthy that some sensors utilized in various devices have faced challenges related to biocompatibility, thereby limiting their in vivo applications, even though there are instances such as contact lens [35, 36], where biocompatible electronic glucose sensors have been effectively developed and utilized. Continuous monitoring of tear fluids using electronic glucose sensors is a popular method, but the sensors used in these devices are generally not biocompatible, limiting their in vivo applications. Additionally, electronic drift in sensor response must be frequently calibrated with fresh blood samples [37]. Electronic glucose systems can also be bulky and complex, and may require excessive electrical components, indicating the need for improvement in device structure and measurement techniques [38].

Optical detection techniques such as surface plasmon resonance (SPR) and fluorescence resonance energy transfer (FRET) have been widely studied for measuring tear glucose concentration [39]. These techniques rely on color changes based on optical indicators that respond to the activation of fluorescently labeled enzymes by glucose. However, this approach may not be suitable for monitoring tear glucose levels due to several limitations. First of all, an additional high-power light source is required to consistently measure the tear glucose concentration in contact lenses, which may cause pain and discomfort to patients due to eye irritation [26, 40–43]. Additionally, the measurement accuracy and clinical practicality of these techniques can easily be hampered by their high sensitivity to motion, the requirement for a lengthy calibration process, and the short lifetime of fluorophores due to degradation. These limitations lead to inefficiency and lack of suitability of optical techniques for monitoring tear glucose levels, despite their potential in vivo and in situ benefits.

In this study, we developed a clinically feasible sucking disk-type (SD) strip biosensor containing a sensing paper coated with cerium oxide nanoparticles (CNPs) and glucose oxidase (GOx). This SD strip biosensor can contact the inferior palpebral conjunctiva (IPC) to collect tears without causing eye irritation, facilitating a brief-use, disposable format that enables individuals to perform self-monitoring of glucose levels with ease. The SD strip biosensor produces a distinctive color change within a glucose concentration range of 0 ~ 2.5 mM, which encompasses tear glucose levels [44], this SD strip biosensor provides immediate, discernible feedback without necessitating any high-power light sources or complex electrical components. Furthermore, with optimized imaging systems and processing algorithms, we ensure maximal measurement accuracy, reaffirming its applicability and reliability for self-diagnosis. In particular, experimental validation performed using this SD strip biosensor system with tear samples from diabetes-inducing rabbits in vivo, and the demonstrated correlation between blood and tear glucose levels, underscore its clinical feasibility and potential as a user-friendly, disposable diagnostic tool.

## Materials and methods

### Materials

Whatman filter paper, cerium (III) nitrate hexahydrate ((Ce(NO3)3·6H2O), sodium hydroxide (NaOH), aminopropyltriethoxysilane (APTS), glucose oxidase, D-( +)-glucose, fluorescein sodium, and phosphate-buffered saline (PBS) were purchased from Sigma Aldrich (St. Louis, MO, USA). All solutions were prepared with ultrapure distilled, deionized water. Materials for 3D printing (VisiJet M3 Crystal) were purchased from 3D Systems (Rock Hill, SC, USA). A glucometer was purchased from Roche Diagnostics (Mannheim, Germany). Ketamine and xylazine were purchased from Yuhan (Seoul, Korea) and Bayer (Leverkusen, Germany), respectively.

### Preparation of the CPNs/APTS/GOx-treated sensing paper

In brief, 1.736 g Ce(NO_3_)_3_ was added to 128 mL of NaOH solution (0.4 g NaOH dissolved in 128 mL of distilled water), and the solution was stirred for 48 h. The white precipitate formed after stirring was washed several times with distilled water and collected. The filter paper was shaken in a colloidal CNP solution at several concentrations (0.5, 1, 3, 5, and 10%) for 10 min to ensure that CNP was sufficiently adsorbed. The CNPs-adsorbed filter paper was dried in a 60 °C oven for 1 h. The adsorbed CNPs were stabilized through silicone coating. APTS forms a covalent bond with the hydroxyl group on the filter paper and CNP surface and is coated with silicone. The paper was shaken in a 5% APTS ethanol solution for 10 min and dried in a 60 °C oven for an additional 10 min [45]. Finally, GOx was applied at 11.43 U/cm^2^ and dried at room temperature to obtain the CPNs/APTS/GOx-treated sensing paper.

### Fabrication of sucking disk-type (SD) strip biosensor

The SD strip biosensor was designed to contain the sensing paper, onto which tear fluid can be collected and delivered. Thus, the SD strip biosensor was composed of three distinct parts connected in series: tip, channel, and reaction chamber. The tip was rounded with a 3-mm diameter, and an opening was made to form the channel (W × H × L: 2 mm × 0.5 mm × 0.3 mm) for tear infiltration. At the end of the channel, the reaction chamber (W × H × L: 1 mm × 0.5 mm × 0.5 mm) was shaped to just fit the sensing paper. A schematic image of a strip-type sensor was drawn with Solidworks (Dassault Systèmes SOLIDWORKS Corp., Waltham, MA, USA). We fabricated the SD strip biosensor with a 3D printer (Projet 3500 HD MAX, 3D Systems, Rock Hill, SC, USA) using VisiJet M3 Crystal (3D Systems, Rock Hill, SC, USA), which is a certified material with known biocompatibility (United State Pharmacopeia Class VI). The illumination model was constructed with an LED spotlight and diffused optics from Edmund Optics. The optical system was constructed with a Sony IMX-128-(L)-AQP CMOS Sensor (Sony Corp., Tokyo, Japan) and objective lens (Edmund Optics, Blackwood, NJ, USA). We removed the shadow and background noise using the algorithm and obtained a color value independent of illuminance through normalization.

### Characterization of the sensing paper

The surface of the sensing paper was characterized using a scanning electron microscope (SEM) (NOVA NANO SEM). X-ray photoelectron spectroscopy (XPS) was used to assess the surface atomic composition of the sensing paper. In addition, energy dispersive spectroscopy (EDS) mapping was used to confirm the distribution of each element on the sensing paper. In particular, it was analyzed through ICP-MS (NexION 350D) to confirm whether CNPs were present at similar levels in each sensing paper. The sensing paper was prepared in a circular shape with a diameter of 6 mm, and after hydrofluoric acid pretreatment, sampling was performed with the same weight and then analyzed. As a control, filter paper was also produced in a circular shape with a diameter of 6 mm and then analyzed. To confirm that GOx was stably attached to the surface of the sensing paper through an immunofluorescence assay, GOx was determined to be evenly distributed through visualization with MATLAB (Mathworks Inc., Natick, MA, USA). To detect glucose in tears using the sensing paper, different glucose concentrations (0, 0.5, 1, and 2.5 mM) were used to confirm the actuation. A 10 μL sample of glucose solution was dropped onto a circular sensing paper with a diameter of 6-mm.

### In vitro evaluation of the SD strip biosensor

To test the sensing paper's reliability to be loaded into the SD strip biosensor, we compared normalized b values before and after loading. The sensing paper was cut into 1 mm × 0.5 mm strips, attached to a petri dish, and treated with 0.5 μL of glucose solutions with concentrations (0 ~ 1.2 mM). On the other hand, the 0.5 mm^2^ piece of the sensing paper was loaded in the reaction chamber of the SD strip biosensor, and 0.5 μL of glucose at concentrations over the same range (0 ~ 1.2 mM) was injected into the channel. After the sensing paper reacted with the glucose solutions for less than 15 min, the yellow color density changed. Then, we acquired the image of the sensing paper with an optical vision system, and the color values of these images were obtained with image processing algorithms.

### In vivo evaluation of SD strip biosensor

Male New Zealand white rabbits (3.0 ~ 3.5 kg body weight) were used in this study to evaluate the correlation between tears and blood glucose levels. The experimental protocol was approved by the Institutional Animal Care and Use Committee (IACUC No. 15–0285-C1A0) at the Biomedical Research Institute of Seoul National University Hospital. To induce diabetic condition in rabbits, we intentionally elevated blood glucose levels via subcutaneous injection of a cocktail of 15 mg/kg ketamine and 5 mg/kg xylazine, as reported in previous studies [37, 46, 47]. At 40 and 80 min after the first injection, a cocktail of 7.5 mg/kg ketamine and 2.5 mg/kg xylazine was administered as boosters, respectively. We measured the glucose levels both in tears and in blood at 15, 22, 30, 45, 60, and 90 min after the first injection of anesthesia. To measure tear glucose level, the tip of the SD strip biosensor was contacted to the IPC of a rabbit eye for 20 s to collect the tear, which infiltrated into the reaction chamber of the SD strip biosensor. In an optical system constructed in the same environment as in vitro, the acquired image and algorithms were used to obtain normalized b values. At the same sampling times, blood was collected from the rabbit ear vein and measured with a commercially-available glucometer (ACCU-CHEK Performa, Roche Diagnostics, Mannheim, Germany). After multiple applications of the SD strip biosensor, we examined any possible tissue damage to the IPC of the rabbit eyes [46, 48]. To this end, a fluorescein solution (0.25% w/v in PBS, 5 μL) was administered topically to the rabbit eye. After 5 min, the eye was thoroughly washed with normal saline. We then obtained a fluorescent image of the IPC, using excitation (475 nm) and emission (542 nm) filters (Thorlabs, Newton, NJ, USA).

### Optical monitoring system and normalization

When a glucose solution reacts in the SD strip biosensor, the yellow intensity of the sensing paper changes at a visible wavelength of about 570 nm. For measuring yellow, we used a white LED light source. The disadvantage of non-uniform intensities of the LED light with a Gaussian distribution was overcome using diffused optics. For color detection, the objective lens was used to enlarge the image of the sensing paper in the SD strip biosensor to × 15 and acquire the image through the integrated digital camera on the microscope. When the images were transferred to a computer, we obtained color values for the sensing paper through the colorimetric analysis algorithm. Because the color values (RGB) include the brightness of the illumination when capturing an image, they vary depending on illumination. Thus, chromaticity values (*ρ*) independent of brightness are obtained with the following normalization formula:

$$\rho (r,g,b)=\frac{R,G,B}{R+G+B}$$

where *R, G,* and *B* represent the intensity of the red, green, and blue color intensity values on the images, respectively.

### Statistics

All data were presented as mean ± S.E.M (standard error of the mean). Statistical analysis was evaluated by Student’s t-tests or one-way analysis of variance (ANOVA). P-value less than 0.05 was considered a statistically significant value. GraphPad Prism (version 8.3.0; GraphPad Software Inc., San Diego, CA, USA) was used for statistical analyses.

## Results

### Design and characterization of CNPs/APTS/GOx-treated sensing paper

**Fig. 1:**
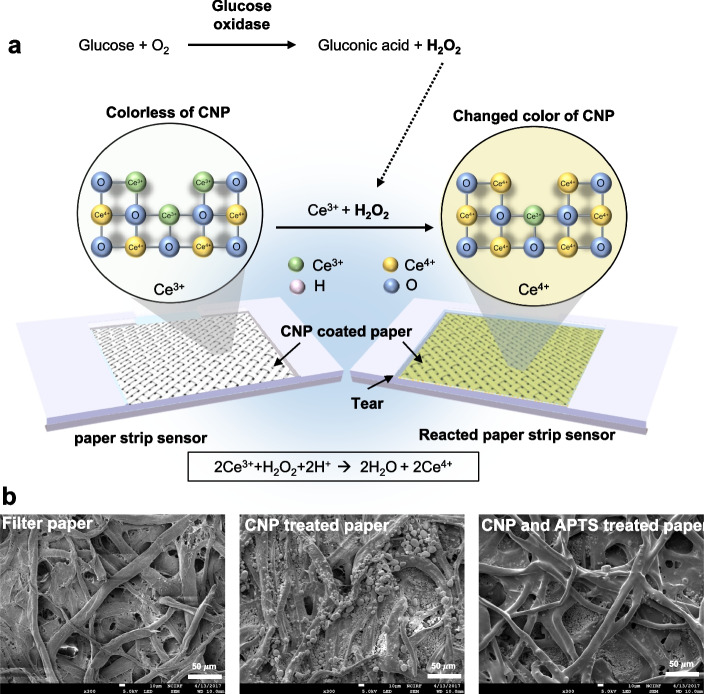
Schematic illustration of the sensor paper treated with cerium oxide nanoparticle (CNP), 3-aminopropyltriethoxysilane (APTS), and glucose oxidase (GOx). **a** Reaction scheme of the immobilized CNP, GOx, and glucose on the sensing paper. The sensing paper color is changed in response to tear glucose concentration from colorless Ce^3+^ (before reaction) to yellow Ce^4+^ (after reaction) due to the oxidation of cerium ions. **b** Captured scanning electron microscopy (SEM) images of filter paper, filter paper treated with CNPs, and filter paper treated with CNPs/APTS

As a glucose sensing material, we used cerium oxide nanoparticles (CNPs) to measure tear glucose concentration after loading on a strip-type sensor. CNPs have been widely used in industry and have recently been studied in biotechnology because of their catalytic activity and low toxicity [49]. CNPs have both Ce^3+^ and Ce^4+^ on their surface, and oxygen vacancy because Ce^3+^ plays an important role in the redox potential of CNPs [50]. When Ce^3+^ oxidizes to Ce^4+^, the colorless CNPs are switched to deep yellow-colored CNPs according to various concentrations of H_2_O_2_ and glucose (Fig. 1 and Fig. S1). This feature provides colorimetric information that can be obtained easily and relatively cheaply through conventional optical measurements [51, 52]. As a result, CNPs have been studied as a sensing material [53, 54]. We characterized the CNPs with high-resolution transmission electron microscopy (HR-TEM) images, suggesting that the synthesized CNPs (6 nm in diameter) had a unique lattice structure on their surface (Fig. S2). The surface of CNPs-treated sensing paper was visualized by using scanning electron microscopy (SEM) (Fig. 1b and Fig. S3).

**Fig. 2:**
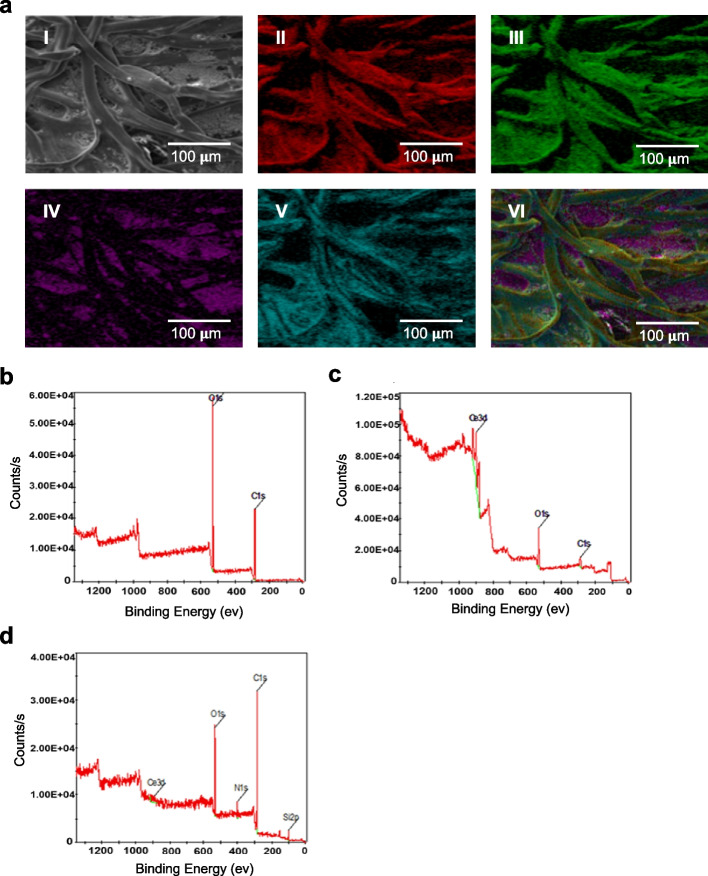
X-ray photoelectron spectroscopy (XPS) characterization and energy dispersive spectroscopy (EDS) mapping with scanning electron microscopy (SEM) of sensing paper treated with CNP and APTS. **a**-I SEM images of CNP- and APTS-treated filter paper. **a**-II** – a**-VI exhibit the element mapping of carbon (red, II), oxygen (green, III), cerium (purple, IV), and silicon (cyan, V), in that order. **a**-VII Merged EDS maps of CNP- and APTS-treated filter paper. **b** XPS spectra of filter paper made of cellulose fiber. **c** XPS spectra of CNP-treated filter paper. The spectra represented Ce3d peak, meaning CNP. **d** XPS spectra of CNP and APTS-treated filter paper. The spectra represented Si2p peak, meaning APTS

**Fig. 3:**
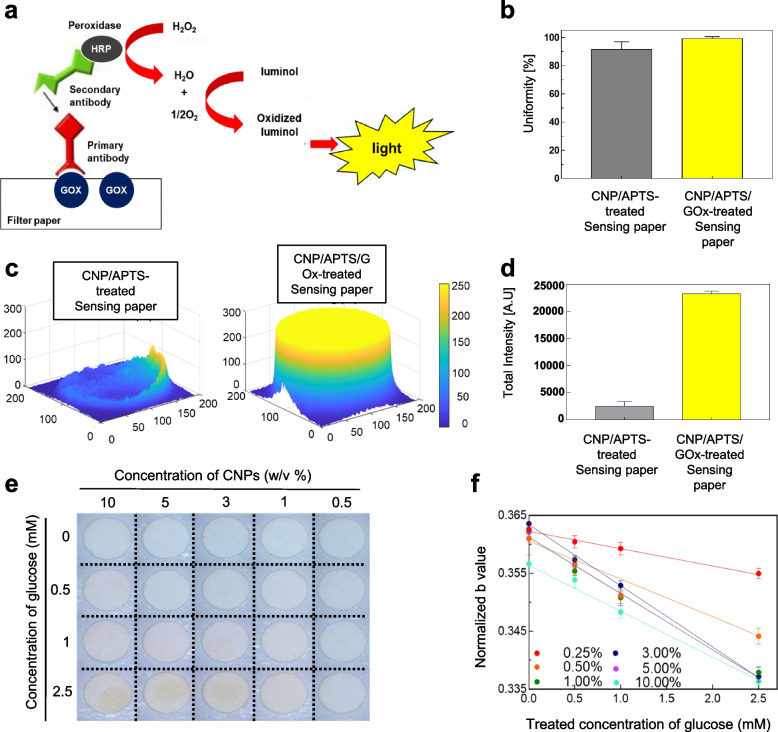
Characterization of the CNPs/APTS/GOx-treated sensing paper. **a** Schematic diagram of an immunofluorescence assay to check the uniform distribution of GOx on the sensing paper. The primary antibody of GOx and secondary antibody were reacted with the sensing paper. **b** Calculated uniformity to verify the uniform distribution of GOx. **c** The MATLAB 3D image to plot the uniformity of CNPs/APTS-treated paper and CNPs/APTS/GOx-treated sensing paper. **d** The total intensity to confirm the uniform distribution of CNPs/APTS-treated paper and CNPs/APTS/GOx-treated sensing paper. **e** Optimization of the concentration of CNPs (0.5, 1, 3, 5, and 10 w/v%) at different glucose concentrations (0, 0.5, 1, and 2.5 mM) for optimal sensitivity of glucose. **f** Linear regression curves of normalized b value from (**e**) result. Data were presented as the mean ± standard deviation (*n* = 5)

To characterize the sensing paper, we analyzed the respective elemental composition and chemical state of the paper surface by using X-ray photoelectron spectroscopy (XPS) and energy dispersive spectroscopy (EDS) mapping (Fig. 2). We performed EDS mapping of the CNPs and 3-aminopropyltriethoxysilane (APTS)-treated filter paper (Figs. S4–S6). Because the filter paper was made of cellulose fiber, the O1s and C1s peaks appeared in the XPS spectrum (Fig. 2a-I, II, b and Fig. S7). After treatment of CNP solution (3% w/v), the Ce3d peak appeared in XPS spectra (Fig. 2a-IV, c and Fig. S8). We treated APTS to stabilize the CNPs adsorbed on the filter paper. APTS reacts with the hydroxyl groups present on the surfaces of the filter paper and CNP to form a covalent bond. After further treatment of APTS, new peaks corresponding to N1s and Si2p appeared (Fig. 2a-V, d and Fig. S9). These XPS and EDS mappings indicate that CNPs and APTS were broadly coated on the whole surface area of the sensing paper. Additionally, to determine whether there was a change in the color reaction of CNPs covalently bonded with APTS, the color development reaction was checked before and after APTS treatment. As a result, there was no significant change in the color reaction before and after APTS treatment (Figure S10). After the treatment of CNPs/APTS on the surface of the sensing paper, the glucose oxidase (GOx) enzyme was finally treated onto the CNP/APTS-treated sensing paper without any chemical cross-linking. Immunofluorescence assay was used to demonstrate the stable immobilization of GOx on the CNP/APTS-treated sensing paper (Fig. 3a). To quantitatively analyze GOx treatment on the sensing paper, we developed image processing algorithms to extract the regions of interest (ROI) within the image (Fig. S11). The quantified uniformity, illustrated by a standard deviation of 90.5 ± 0.03% for CNPs/APTS-treated paper and 99.2 ± 0.99% for CNPs/APTS/GOx-treated sensing paper in Fig. 3b, underscores the high uniformity and replicability in our fabrication process, demonstrating the reliable distribution of CNPs and GOx throughout the sensing paper. In addition, the 10-fold higher fluorescence signal with 23,322 ± 400.3 A.U was equally detected on the whole area of CNPs/APTS/GOx-treated sensing paper than CNP/APTS treated sensing paper with 2,512.8 ± 712.8 A.U (Fig. 3c and d), suggesting that a higher density of GOx enzyme was consistently and evenly coated on the entire area of CNPs/APTS-treated sensing paper. The uniform distribution of GOx on the sensing paper played an important role in the accurate and reliable detection of the target molecule in the sample, thus ensuring the reproducibility of the sensing performance. Next, we optimized the concentration and volume of CNPs to maximize the sensitivity of various concentrations of glucose on the sensing paper. The color change was not significant at low CNP concentrations (0.5% w/v), whereas the base color of the paper was too yellow to detect a change in color at high CNP concentrations (10% w/v) (Fig. 3e and g). ICP-MS analysis was performed to quantify the CNPs present in the sensing paper. The sensing paper was sampled with the same weight (15 g) after hydrofluoric acid pretreatment, and the amount of CNPs present in it was measured. As a result, the average and standard deviation of the amount of CNP present in the sample were measured to be 6453.7 ppb and 416.2 ppb, respectively (Table S1). Based on this result, the uniformity is 92.4%, which means that the CNPs present in the sensing paper are evenly distributed. Additionally, our study highlighted the importance of macro-scale uniformity in CNP distribution. We utilized an RGB camera to capture the entire sensor area, effectively balancing practical application with in-depth analysis. Despite possible height variations due to the fibrous nature of the filter paper, our sensor was designed to reliably detect uniform signals across the entire area at a macro-scale level in Fig. 3c. To quantitatively analyze the color change, we optimized the image processing algorithm (Fig. S12a). This algorithm could automatically calculate the degree of color change, which is proportional to the concentration of glucose present in the sample. Because the complementary color (blue) of the reflected color (yellow) is more sensitive than other reflected green and red colors, the glucose concentration was analyzed as blue color with the normalization formula (Fig. S12b). By using this image processing algorism, the optimal concentration of CNP was 3% (w/v) to detect glucose concentration on the CNPs/APTS/GOx-treated sensing paper, showing the largest and most significant slope in the linear regression graph (*R*^2^ = 0.998) (Fig. 3f and h).

### Fabrication of sucking disk-type (SD) strip biosensor with the CNPs/APTS/GOx-treated sensing paper

**Fig. 4:**
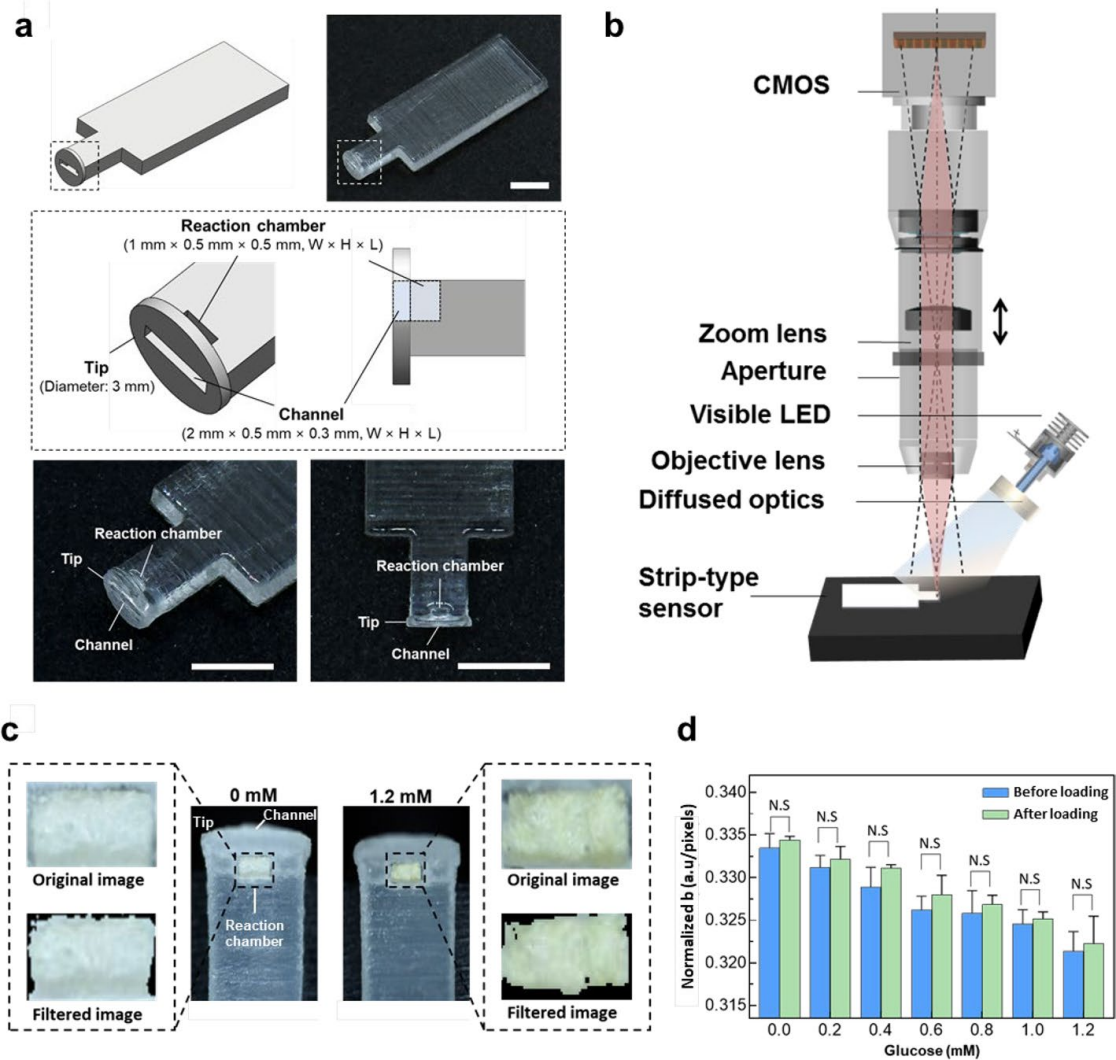
Actuation of sucking disk-type (SD) strip biosensor embedded with CNPs/APTS/GOx-treated sensing paper. **a** Schematic and optical images image of the sucking disk-type (SD) strip sensor. The CNPs/APTS/GOx-treated sensing paper was inserted in the reaction chamber. Scale bar = 5 mm. **b** Schematic image of the optical system to capture the colorimetric images of the CNPs/APTS/GOx-treated sensing paper in the reaction chamber with a tiny window to the SD strip biosensor. **c** Real image (original) and normalized image (filtered) of the sensing paper in the reaction chamber of SD strip biosensor before and after treatment of glucose solution (1.2 mM). The normalized image was obtained with an image processing algorithm. **d** Correction test for comparison of sensing paper performance. We monitored color variation by calculating the normalized blue (normalized b) value between sensing paper before and after being loaded into the SD strip biosensor. Data were presented as the mean ± standard deviation (*n* = 5). NS: No significant difference

The design of the strip-type biosensor needs to be well-suited for collecting and delivering tear fluid to the sensing paper. To properly collect and deliver tear fluid to the sensing paper, we designed the sucking disk-type (SD) strip biosensor consisting of a tip, channel, and reaction chamber connected in series, which could easily be manufactured by using a 3-dimensional printer device (Fig. 4a). This is important because any damage to the eye surface can cause discomfort and may lead to complications such as infection or inflammation [43, 55, 56]. The rounded sucking disk tip can help to distribute the pressure evenly during the collection of tears at the inferior palpebral conjunctiva (IPC) without any eye irritation. In addition, the rectangular-shaped channel is adopted to effectively suck tears by increasing capillary pressure [57]. Next, the prepared sensing paper was cut to insert into the reaction chamber of the SD strip biosensor. After that, we tested the color changes in the reaction chamber of the SD strip biosensor before and after the treatment of glucose (1.2 mM, 0.5 μL) (Fig. 4c). In this process, we newly manufactured the tailored optical system to exactly analyze the color change of CNPs/APTS/GOx-treated sensing paper in the reaction chamber with a tiny window (1 mm × 0.5 mm) (Fig. 4b). Also, we optimized the normalized image process to quantitatively analyze the color change in the reaction chamber with a tiny window (Fig. S12a, Fig. 4c, and Fig. S13). The original and normalized image could reflect the different glucose concentrations. Finally, we compared the color variation of the sensing paper before and after being loaded into the SD strip biosensor at various concentrations of glucose (0.5 μL) (Fig. 4d). In both groups, they were not significantly different, both showing that the normalized b values proportionally decreased as glucose concentration proportionally increased (*R*^2^ = 0.9716). This correlation test showed that the sensing papers had an almost consistent profile, suggesting that the function was unchanged after the sensing paper was loaded into the SD strip biosensor. In addition, we have confirmed the color variation of SD strip biosensor at low glucose concentrations (0.001, 0.005, 0.01, and 0.1 mM). Notably, a significant colorimetric change was observed at 0.1 mM, as confirmed by our optical monitoring system (*p* < 0.05 mM), as depicted in Supporting Information Fig. S14.

### In vivo evaluation of the SD strip biosensor in a diabetic rabbit model

**Fig. 5:**
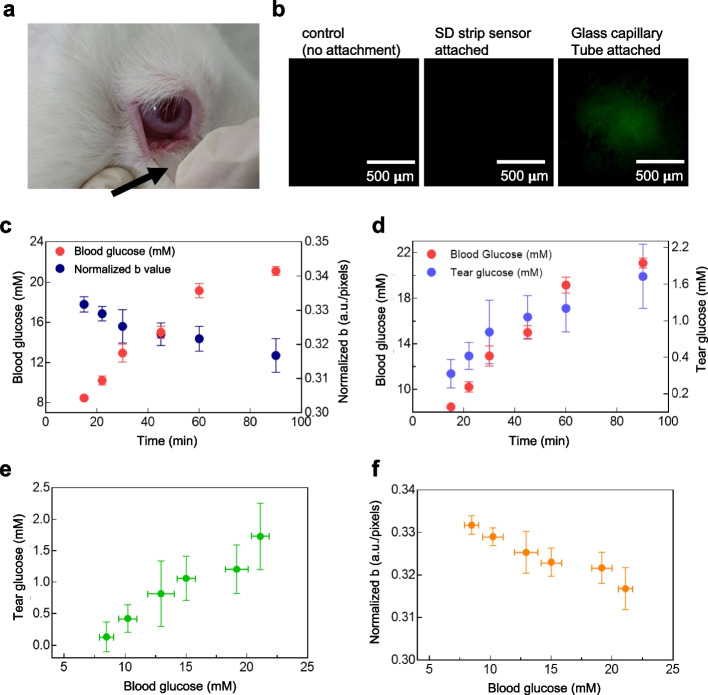
Blood glucose prediction by using measurement of tear glucose levels with the SD strip biosensor. **a** Touch of SD strip biosensor in the inferior palpebral conjunctiva (IPC) of a diabetic rabbit model. Arrow: SD strip biosensor. **b** Monitoring surface damage to the eye during tear collection with a glass capillary tube or SD strip biosensor. Eye damage was visualized after instillation into the rabbit IPC with 5 μL of 0.25% w/v fluorescein sodium solution. Fluorescein image: eye damage. Fluorescent region indicates eye tissue damage. **c** The comparison between the normalized b values and blood glucose concentration at each time point. At each sampling time, five rabbits were tested and a single measurement was performed for each animal. Data were presented as the mean ± standard deviation (*n* = 5). **d** The comparison between the tear glucose and blood glucose concentration at each time point. Data were presented as the mean ± standard deviation (*n* = 5). **e** A linear correlation between blood and tear glucose levels. Data were presented as the mean ± standard deviation (*n* = 5). **f** A linear correlation between blood glucose levels and normalized b values. Data were presented as the mean ± standard deviation (*n* = 5)

The actuation of the SD strip biosensor was evaluated in the inferior palpebral conjunctiva (IPC) of a diabetic rabbit model (Fig. 5a, arrow). When the SD strip biosensor was in contact with the IPC, the tear was successfully absorbed through the channel and diffused into the reaction chamber with the CNPs/APTS/GOx-treated sensing paper. The entire working process of the SD strip biosensor shown in Movie S1. To evaluate the damage to the eye surface after the in vivo experiments, the IPC of the eye of the rabbit was stained with fluorescein (Fig. 5b). The experiment revealed no staining of tissue, indicating no IPC damage even after multiple contacts with the SD strip biosensor (Fig. S15a). In contrast, when the IPC was contacted one time with a glass capillary tube that is conventionally used for tear collection, a fluorescence-stained region was clearly observed due to tissue damage [46, 56]. Quantitative measurements of the damaged area with the developed image processing algorithm showed a significant difference between the glass capillary tube and the SD strip biosensor (Fig. S1b). In addition, this SD strip biosensor does not require a huge volume of tears because the channel of the SD strip biosensor is designed to possess a high capillary pressure with minimal dead volume (Fig. 4a). The channel of the SD strip biosensor had a rectangular cross-section and was 0.5 mm in height, 2 mm in width, and 0.3 mm in length. The reaction chamber was shaped to just fit the sensing paper at 0.5 mm^2^. In this way, a fixed volume (0.5 µL) of tear fluid could be collected to react with the sensing paper, which is smaller than the total volume of basal tear fluid available in the preocular space [58].

In the diabetes-inducing rabbit animal model, tear fluid was collected within a few seconds and measured at scheduled times using the SD strip biosensor while the blood glucose level was also measured concurrently (Fig. 5c). The normalized b values obtained from each sampled measurement decreased as the blood glucose level increased. When the normalized b value was converted to the tear glucose level through the measurement index described above, there was a strong correlation between tear and blood glucose levels during the diabetes-inducing procedure in rabbits (Fig. 5d) [26, 59]. The glucose elevation profiles of the rabbits in a fasting state were almost consistent for all individuals, with each rabbit reaching glucose levels within a diabetic range [10, 27]. Throughout this range, the SD strip biosensor successfully estimated glucose levels in tears of the diabetic rabbits. As the sensor herein is designated for sampled measurements, the reproducibility of the sensor performance is expected to be maintained within the tested range of blood glucose level. Figure 5e shows the linear relationship between glucose levels in tears and in blood (*R*^2^ = 0.95). Again, a strong correlation was observed between the normalized b value and blood glucose level (Fig. 5f). The observation shows the applicability of the current technique for the management of diabetes and indicates a correlation between tear glucose and blood glucose level as shown in previous studies [26, 59]. While further in vivo validation is needed to improve clinical use, our study demonstrates the potential for safely and accurately measuring a wide range of glucose concentrations in tears by using SD strip biosensor.

## Discussion

This study presents a comprehensive approach to tear glucose monitoring by combining the advantageous properties of cerium oxide nanoparticles, precise immobilization of glucose oxidase, advanced image processing algorithms, and a user-friendly biosensor design. The results underscore the potential for a fully automated and safe tear glucose monitoring system that could significantly contribute to diabetes management in the clinical setting.

The CNPs-based sensing paper developed in this study offers several advantages for glucose sensing applications. The colorimetric response of CNPs to glucose concentration changes provides a simple and visual method for detecting glucose levels. Characterization of the CNPs-treated sensing paper was carried out through a combination of high-resolution transmission electron microscopy (HR-TEM) and scanning electron microscopy (SEM). These techniques provided insight into the unique lattice structure of the synthesized CNPs and visualized the surface of the sensing paper. This characterization not only confirmed the successful synthesis of the CNPs but also validated their uniform distribution on the sensing paper, a pivotal factor for achieving consistent and reliable sensing performance. The functionalization of the sensing paper involved a sequential treatment of CNPs and 3-aminopropyltriethoxysilane (APTS), followed by the immobilization of glucose oxidase (GOx) without the need for chemical cross-linking. The use of immunofluorescence assay provided robust evidence of the stable and uniform immobilization of GOx on the CNP/APTS-treated sensing paper. To quantitatively analyze the GOx distribution, sophisticated image processing algorithms were developed to accurately extract regions of interest (ROIs). The even distribution of GOx across the entire surface of the CNP/APTS-treated sensing paper is a key factor in achieving consistent and reproducible sensing outcomes. The optimization of CNP concentration, determined to be 3% (w/v), exemplifies the intricate balance between color change intensity and sensitivity. The developed image processing algorithm, based on the complementary color (blue) of the reflected color (yellow), provides a robust approach for quantitatively assessing glucose concentrations on the CNPs/APTS/GOx-treated sensing paper. The high linearity achieved (*R*^2^ = 0.998) in the glucose concentration detection range underscores the reliability and accuracy of the developed biosensor. From a clinical perspective, the detection of hypoglycemia holds paramount importance for patients with diabetes mellitus, given the potential for serious complications that often necessitate emergency medical attention. Our benchtop study has demonstrated the technical feasibility of quantifying tear glucose levels below 0.2 mM, with the lowest detection limit being 0.1 mM (Refer to Supporting Information Fig. S14). This observation suggests the potential applicability of our current technique in detecting hypoglycemia.

The sucking disk-type (SD) strip biosensor fabrication, specifically designed to collect and deliver tear fluid to the sensing paper, marks a significant advancement in tear glucose monitoring. The ease of manufacturing this biosensor, using a 3-dimensional printer, provides economic advantages compared to conventional tear glucose biosensors. Importantly, the SD strip biosensor's design considers the well-being of the eye surface, minimizing potential damage and discomfort during tear collection. The successful evaluation of the SD strip biosensor in a diabetic rabbit model further substantiates its potential for practical application. The absence of tissue damage, even after multiple contacts with the biosensor, highlights its safety. The strong correlation observed between tear glucose and blood glucose levels in the rabbit model emphasizes the biosensor's reliability and potential clinical utility.

## Conclusions

In conclusion, we processed the CNPs/APTS/GOx-treated sensing paper. Then the sensing paper was easily mounted in the reaction chamber of the SD strip biosensor manufactured with a 3-dimensional printer. Because the sensing paper and the SD strip biosensor can be manufactured with facile processes, this SD strip biosensor system is economically advantageous compared with other tear glucose biosensors. In addition, this SD strip biosensor system was successfully evaluated with the optimized image algorism in the diabetes-inducing rabbit model. Collectively, this SD strip biosensor system can be in a significant step toward developing a fully automated instrument for tear glucose monitoring in diabetes patients.

### Supplementary Information

**Additional file 1: Movie S1.** The Entire working process of SD strip biosensor. **Table S1.** The concentration of cerium in samples. **Fig. S1.** Color change showing that CNPs reacted with H_2_O_2_ or glucose solution. **Fig. S2.** HR-TEM image with a magnification of ×200,000 of CNPs, showing the distinct structure of the nanoparticles. **Fig. S3.** SEM image of CNPs-treated sensing paper (a) and CNPs/APTS-treated sensing paper. **Fig. S4.** EDS mapping of bare sensing paper. **Fig. S5.** EDS mapping of CNP-treated sensing paper. **Fig. S6.** EDS mapping of CNPs/APTS-treated sensing paper. **Fig. S7.** XPS survey spectra of bare sensing paper. **Fig. S8.** XPS survey spectra of CNPs-treated sensing paper. **Fig. S9.** XPS survey spectra of CNPs/APTS-treated sensing paper. **Fig. S10.** Comparison of normalized b values before and after APTS treatment in CNPs conjugation at the different concentration of H_2_O_2_. N.S indicates no significant difference. **Fig. S11.** The overall process for calculating total intensity and uniformity with an immunofluorescence image (inverted image) of CNPs/APTS-treated and CNPs/APTS/GOx-treated sensing paper. (a) Image processing algorithm to evaluate the CNPs/APTS-treated sensing paper and CNPs/APTS/GOx-treated sensing paper. (b) The overall image process for calculating the effect of GOx treatment on the CNPs/APTS-treated sensing paper. **Fig. S12.** The comparison and image processing between original RGB and normalized rgb image. **Fig. S13.** The overall process to estimate the color change of the sensing paper in the action chamber of SD strip biosensor in response to tear glucose concentration. **Fig. S14.** Assessment of the Limit of Detection (LOD) for Low Glucose Concentrations. **Fig. S15.** Image analysis of eye damage using fluorescent dye after touching the SD strip biosensor to the IPC of the rabbit eye.

## Data Availability

The datasets used and/or analyzed during the current study are available from the corresponding author on reasonable request.

## Abbreviations

SD: Sucking disk-type
CNP: Cerium Oxide Nanoparticle
GOx: Glucose Oxidase
BGST: Blood glucose self-testing
SPR: Surface Plasmon Resonance
FRET: Fluorescence Resonance Energy Transfer
IPC: Inferior Palpebral Conjunctiva
APTS: Aminopropyltriethoxysilane
PBS: Phosphate-Buffered Saline
NaOH: Sodium hydroxide
SEM: Scanning Electron Microscope
HR-TEM: High-resolution transmission electron microscopy
XPS: X-ray Photoelectron Spectroscopy
EDS: Energy Dispersive Spectroscopy
ROI: Regions of Interest
