# Supplementary material for: Chromophoric cerium oxide nanoparticle-loaded sucking disk-type strip sensor for optical measurement of glucose in tear fluid
Source: Biomater Res. 2023 Dec 18;27:135. doi: 10.1186/s40824-023-00469-5 (PMC10729336; doi:10.1186/s40824-023-00469-5)
Supplement: Supplementary file 1 — Additional file 1: Movie S1. The Entire working process of SD strip biosensor. Table S1. The concentration of cerium in samples. Fig. S1. Color change showing that CNPs reacted with H2O2 or glucose solution. Fig. S2. HR-TEM image with a magnification of ×200,000 of CNPs, showing the distinct structure of the nanoparticles. Fig. S3. SEM image of CNPs-treated sensing paper (a) and CNPs/APTS-treated sensing paper. Fig. S4. EDS mapping of bare sensing paper. Fig. S5. EDS mapping of CNP-treated sensing paper. Fig. S6. EDS mapping of CNPs/APTS-treated sensing paper. Fig. S7. XPS survey spectra of bare sensing paper. Fig. S8. XPS survey spectra of CNPs-treated sensing paper. Fig. S9. XPS survey spectra of CNPs/APTS-treated sensing paper. Fig. S10. Comparison of normalized b values before and after APTS treatment in CNPs conjugation at the different concentration of H2O2. N.S indicates no significant difference. Fig. S11. The overall process for calculating total intensity and uniformity with an immunofluorescence image (inverted image) of CNPs/APTS-treated and CNPs/APTS/GOx-treated sensing paper. (a) Image processing algorithm to evaluate the CNPs/APTS-treated sensing paper and CNPs/APTS/GOx-treated sensing paper. (b) The overall image process for calculating the effect of GOx treatment on the CNPs/APTS-treated sensing paper. Fig. S12. The comparison and image processing between original RGB and normalized rgb image. Fig. S13. The overall process to estimate the color change of the sensing paper in the action chamber of SD strip biosensor in response to tear glucose concentration. Fig. S14. Assessment of the Limit of Detection (LOD) for Low Glucose Concentrations. Fig. S15. Image analysis of eye damage using fluorescent dye after touching the SD strip biosensor to the IPC of the rabbit eye. [file 40824_2023_469_MOESM1_ESM.docx]

**Supporting Information**

**Chromophoric cerium oxide nanoparticle-loaded sucking disk-type strip sensor for optical measurement of glucose in tear fluid**

Sijin Park^1,^*, Dong Yeon Nam^2,^*, Hee-Jae Jeon^3,^*, Jae Hoon Han^2,^*, Dawon Jang^1^, Juil Hwang^4^, Yeong-Seo Park^3^, Young-Geun Han^4^, Young Bin Choy^2,5,6,^**, Dong Yun Lee^1,7,8,^**

**Total Supporting Movie (S1)**

Movie S1. The Entire working process of SD strip biosensor.

**Total Supporting Table (S1)**

**Table S1. The concentration of cerium in samples.**

**Total Supporting Figures (S1~S15)**

Fig. S1. Color change showing that CNPs reacted with H_2_O_2_ or glucose solution.

Fig. S2. HR-TEM image with a magnification of ×200,000 of CNPs, showing the distinct structure of the nanoparticles.

Fig. S3. SEM image of CNPs-treated sensing paper (a) and CNPs/APTS-treated sensing paper

Fig. S4. EDS mapping of bare sensing paper.

Fig. S5. EDS mapping of CNP-treated sensing paper

Fig. S6. EDS mapping of CNPs/APTS-treated sensing paper.

Fig. S7. XPS survey spectra of bare sensing paper.

Fig. S8. XPS survey spectra of CNPs-treated sensing paper.

Fig. S9. XPS survey spectra of CNPs/APTS-treated sensing paper.

Fig. S10. Comparison of normalized b values before and after APTS treatment in CNPs conjugation at the different concentration of H_2_O_2_. N.S indicates no significant difference.

Fig. S11. The overall process for calculating total intensity and uniformity with an immunofluorescence image (inverted image) of CNPs/APTS-treated or CNPs/APTS/GOx-treated sensing paper.

Fig. S12. The comparison and image processing between original RGB and normalized rgb image.

Fig. S13. The overall process to estimate the color change of the sensing paper in the action chamber of SD strip biosensor in response to tear glucose concentration.

Fig. S14. Assessment of the Limit of Detection (LOD) for Low Glucose Concentrations.

Fig. S15. Image analysis of eye damage using fluorescent dye after touching the SD strip biosensor to the IPC of the rabbit eye.

**Movie S1**. The Entire working process of SD strip biosensor.


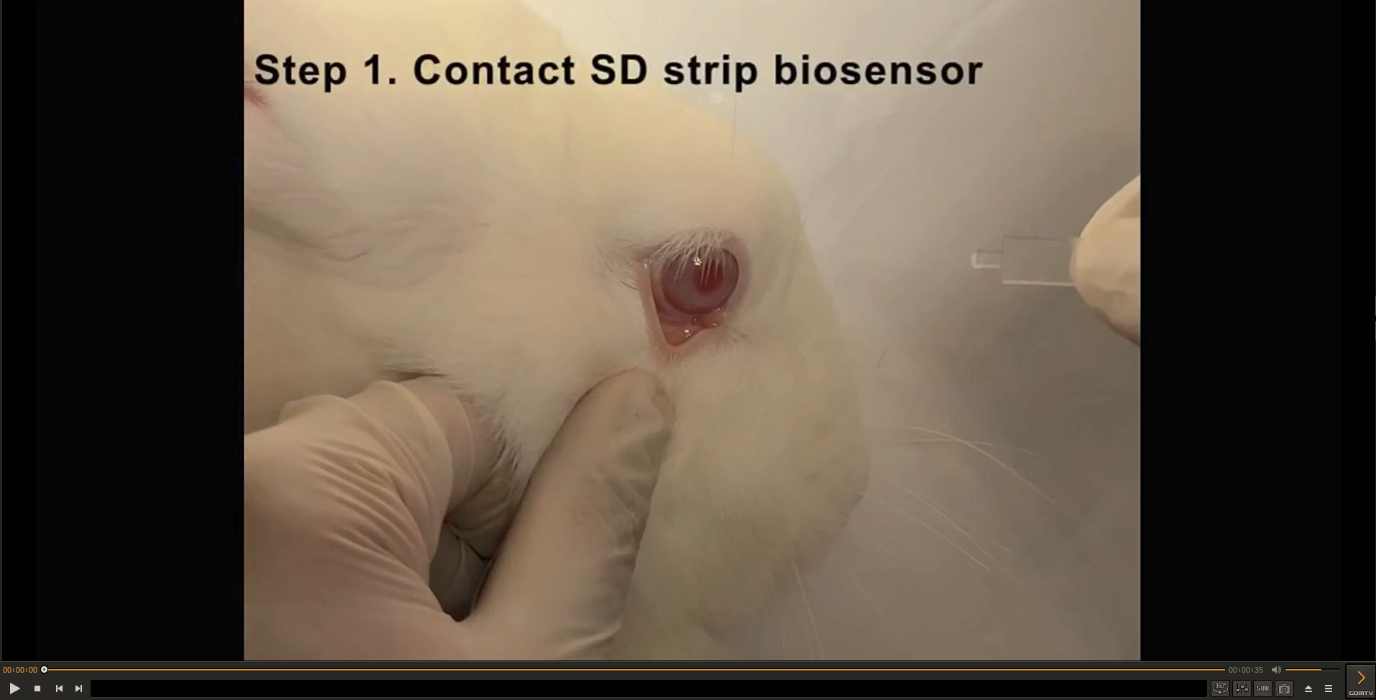


**Table S1. The concentration of Cerium in samples.**

| **sample** | | **Ce (ppb)** | **mean^a^ ± S.E.M^b^** | **Uniformity^c^** |
| --- | --- | --- | --- | --- |
| Filter paper | 1 | N.D. | - | - |
|  | 2 | N.D. |  |  |
|  | 3 | N.D. |  |  |
|  | 4 | N.D. |  |  |
|  | 5 | N.D. |  |  |
| Sensing paper | 1 | 7029.3 | 6453.7 ± 208.1 | 92.4 |
|  | 2 | 6391.0 |  |  |
|  | 3 | 5960.1 |  |  |
|  | 4 | 6822.9 |  |  |
|  | 5 | 6065.4 |  |  |

^a^ mean of samples 1 to 5

^b^ standard error of the mean

^c^ the percentage of the minimum to the mean

**
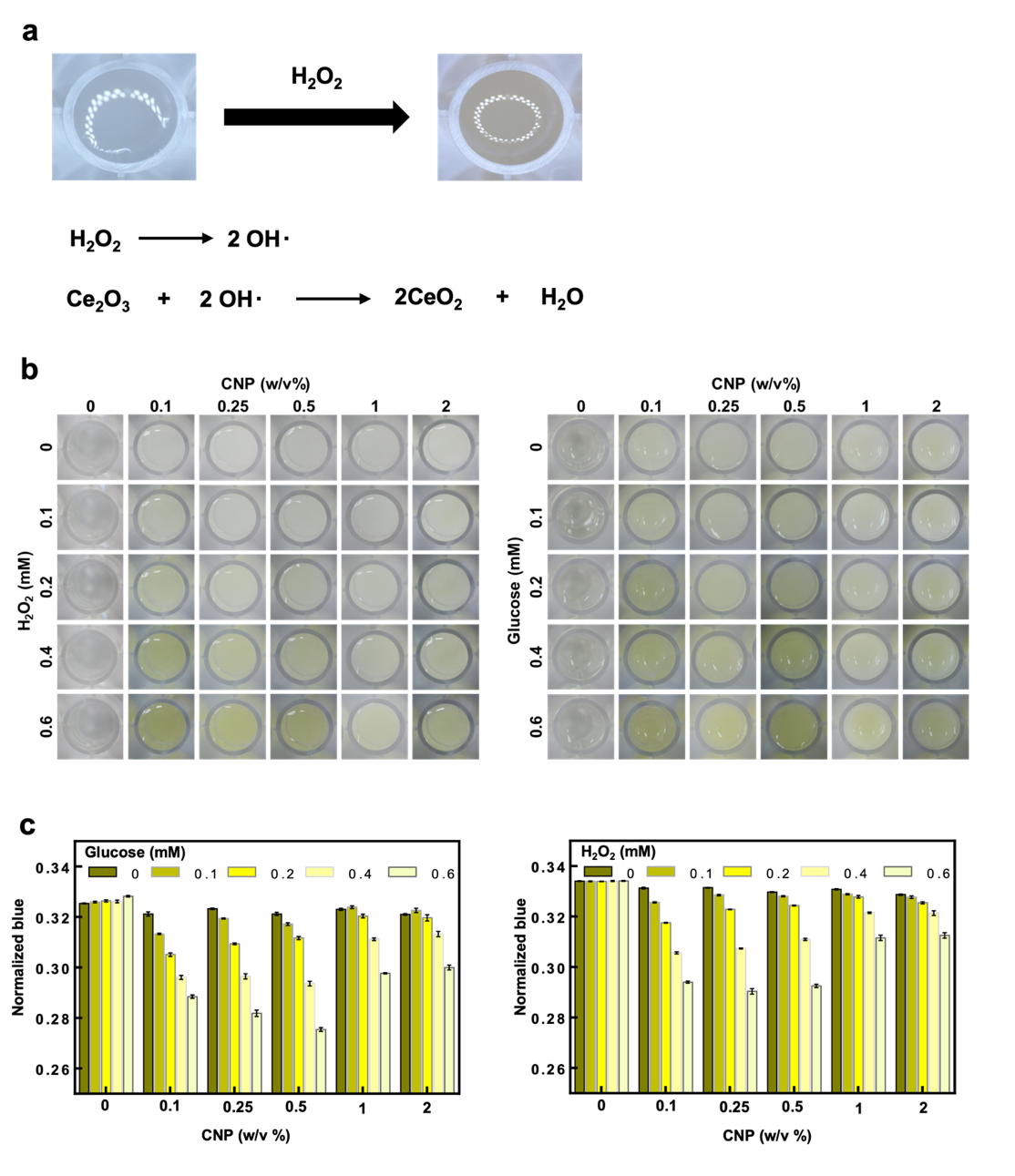
**

**Fig. S1. Color change showing that CNPs reacted with H_2_O_2_ or glucose solution.** **(a)** When H_2_O_2_ (0.6 mM) was treated with CNP solution (0.25 w/v%), the color immediately changed to yellow. This change in color is an inherent property of the CNPs and occurs because free radicals generated by H_2_O_2_ react with the Ce_2_O_3_ state on the surface of the CNPs to produce CeO_2_. **(b)** Color images of different concentrations (0, 0.1, 0.25, 0.5, 1, and 2 w/v%) of CNPs at different concentrations (0, 0.1, 0.2, 0.4, and 0.6 mM) of hydrogen peroxide (H_2_O_2_, left) or glucose (right) in the presence of glucose oxidase (GOx; 100 U/mL). **(c)** Normalized b value in the various concentration of glucose (left) and hydrogen peroxide (H_2_O_2_, right).

**
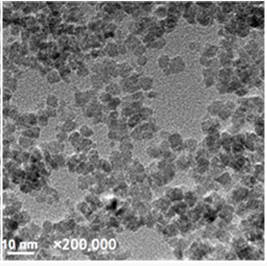
**

**Fig. S2.** **HR-TEM image with a magnification of ×200,000 of CNPs, showing the distinct structure of the nanoparticles**. Scale bar = 10 nm.


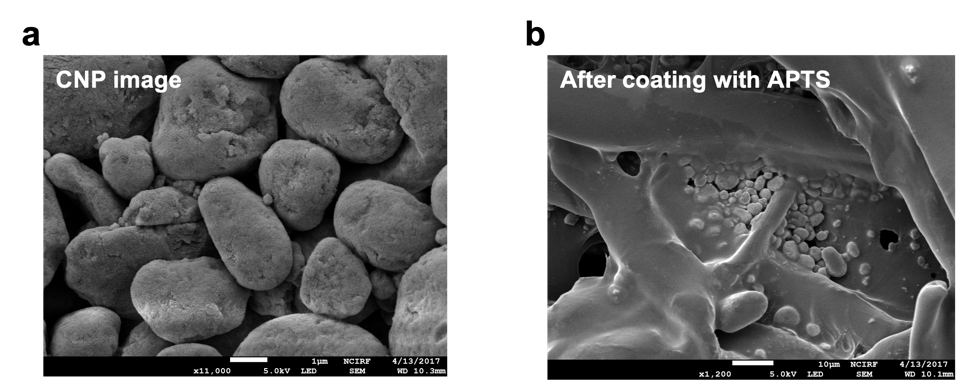


**Fig. S3.** **SEM image of CNPs-treated sensing paper (a) and CNPs/APTS-treated sensing paper.**


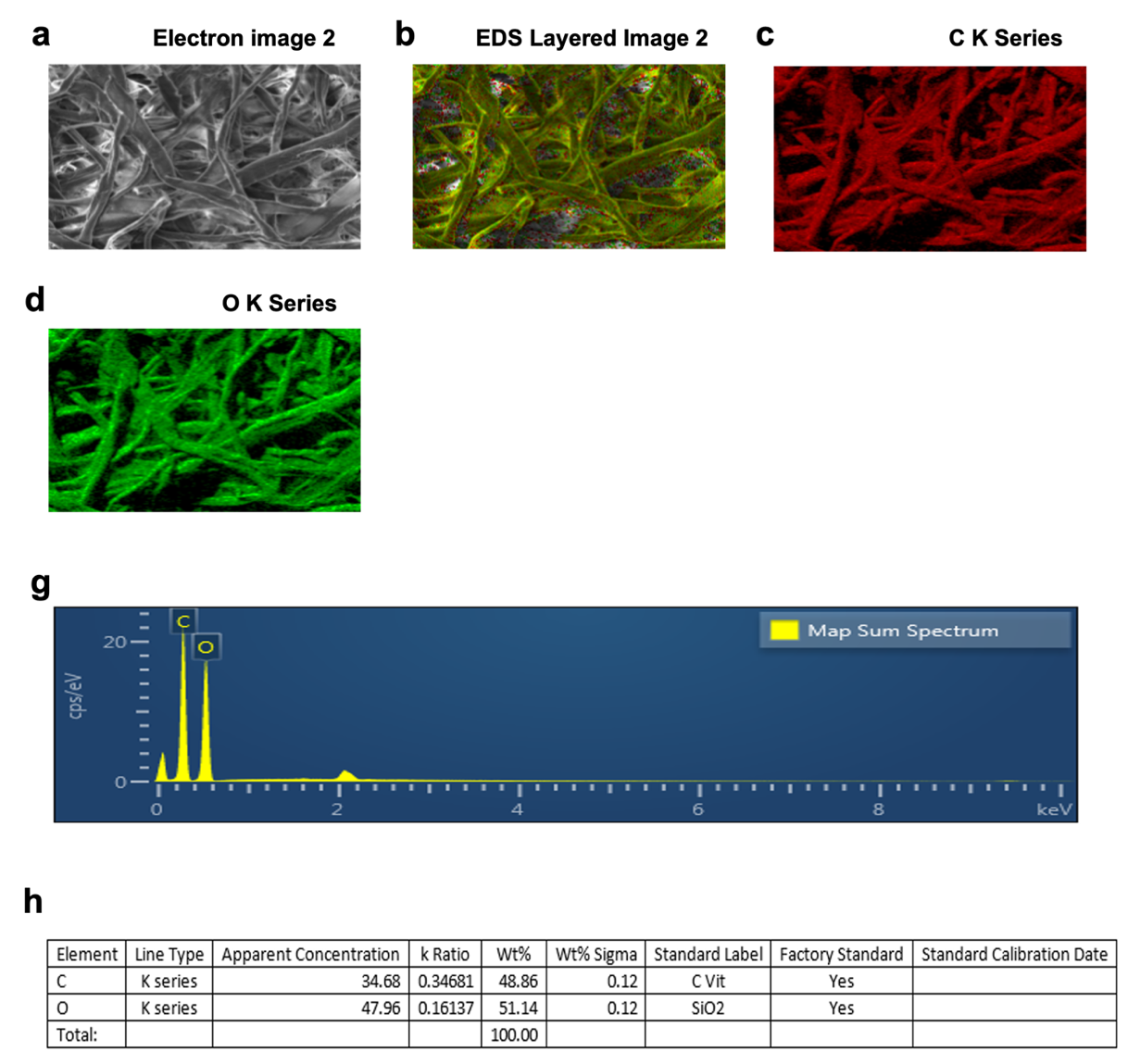


**Fig. S****4**. **EDS mapping of bare sensing paper.** (a) SEM image of bare sensing paper. (b)–(d) Element-sensitive maps of carbon and oxygen. (e) and (f) Map Sum Spectrum and percentages of elements in filter paper.


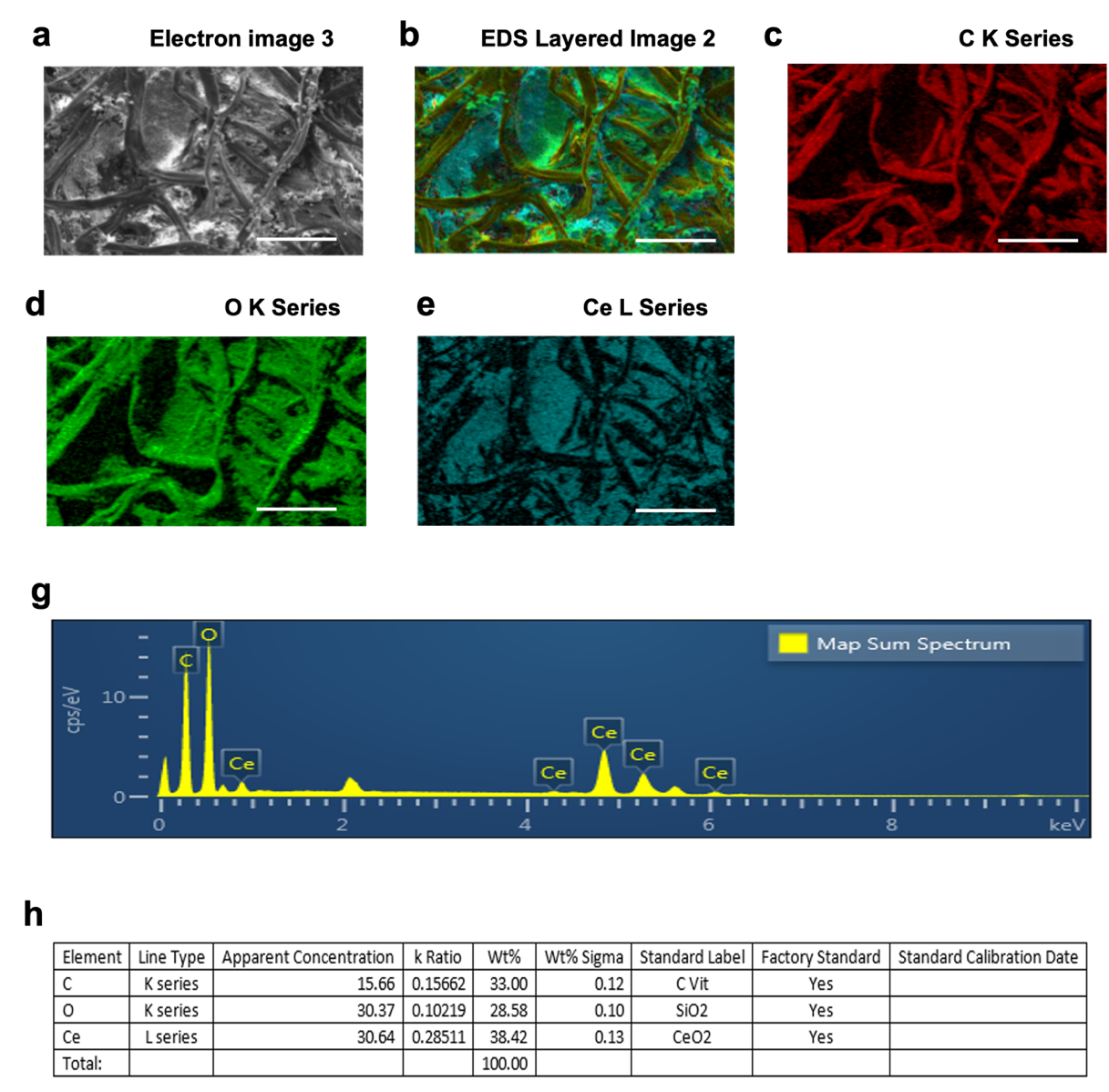


**Fig. S5. EDS mapping of CNP-treated sensing paper.** (a) SEM image of CNPs-treated sensing paper. (b)–(e) Element-sensitive maps of carbon, oxygen, and cerium. (f) and (g) Map Sum Spectrum and percentages of elements in CNPs-treated sensing paper.


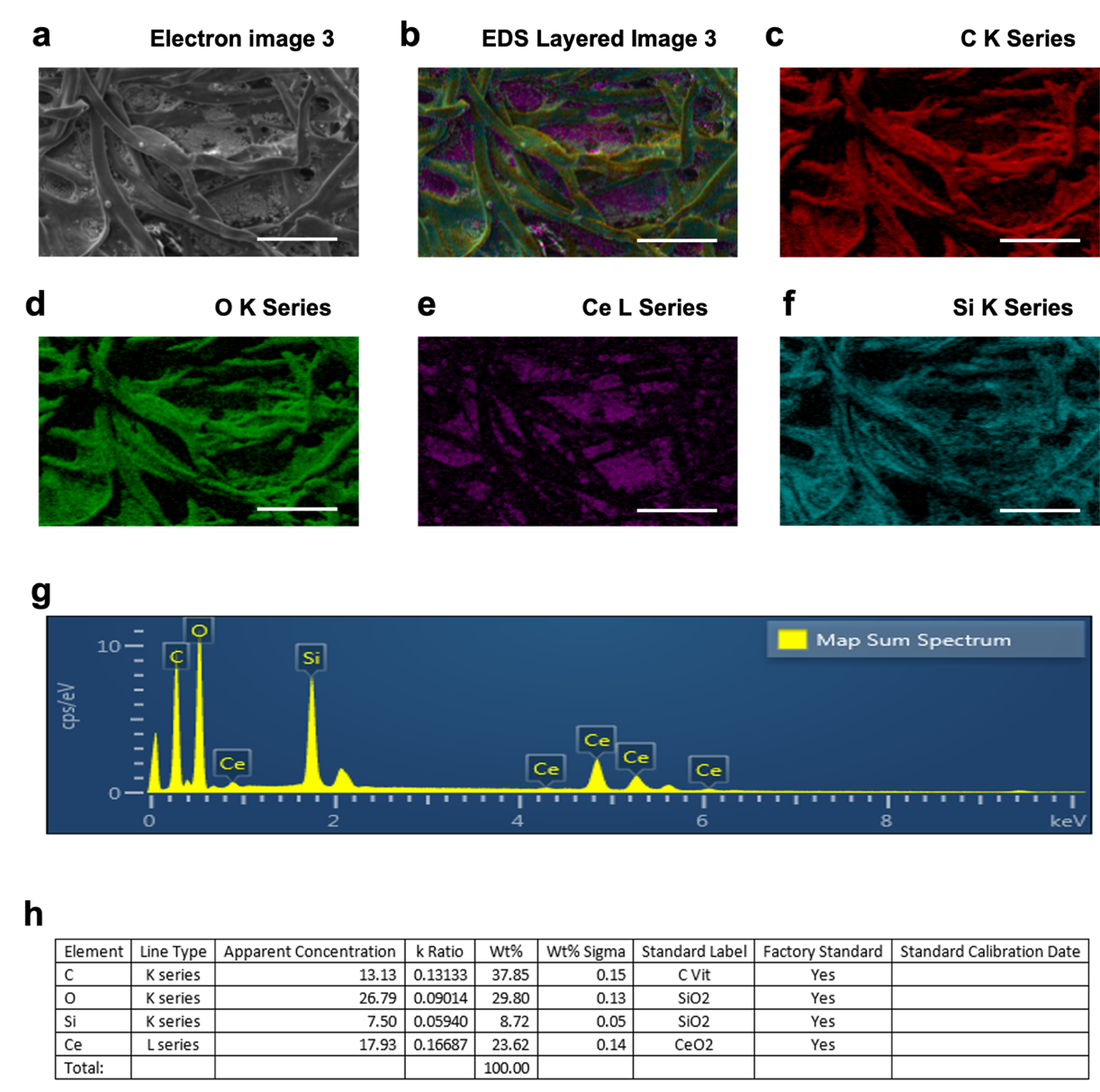


**Fig. S6. EDS mapping of CNPs/APTS-treated sensing paper.** (a) SEM image of CNPs/APTS-treated sensing paper. (b)–(f) Element-sensitive maps of carbon, oxygen, cerium, and silicon. (g) and (h) Map Sum Spectrum and percentages of elements in CNPs/APTS-treated sensing paper.


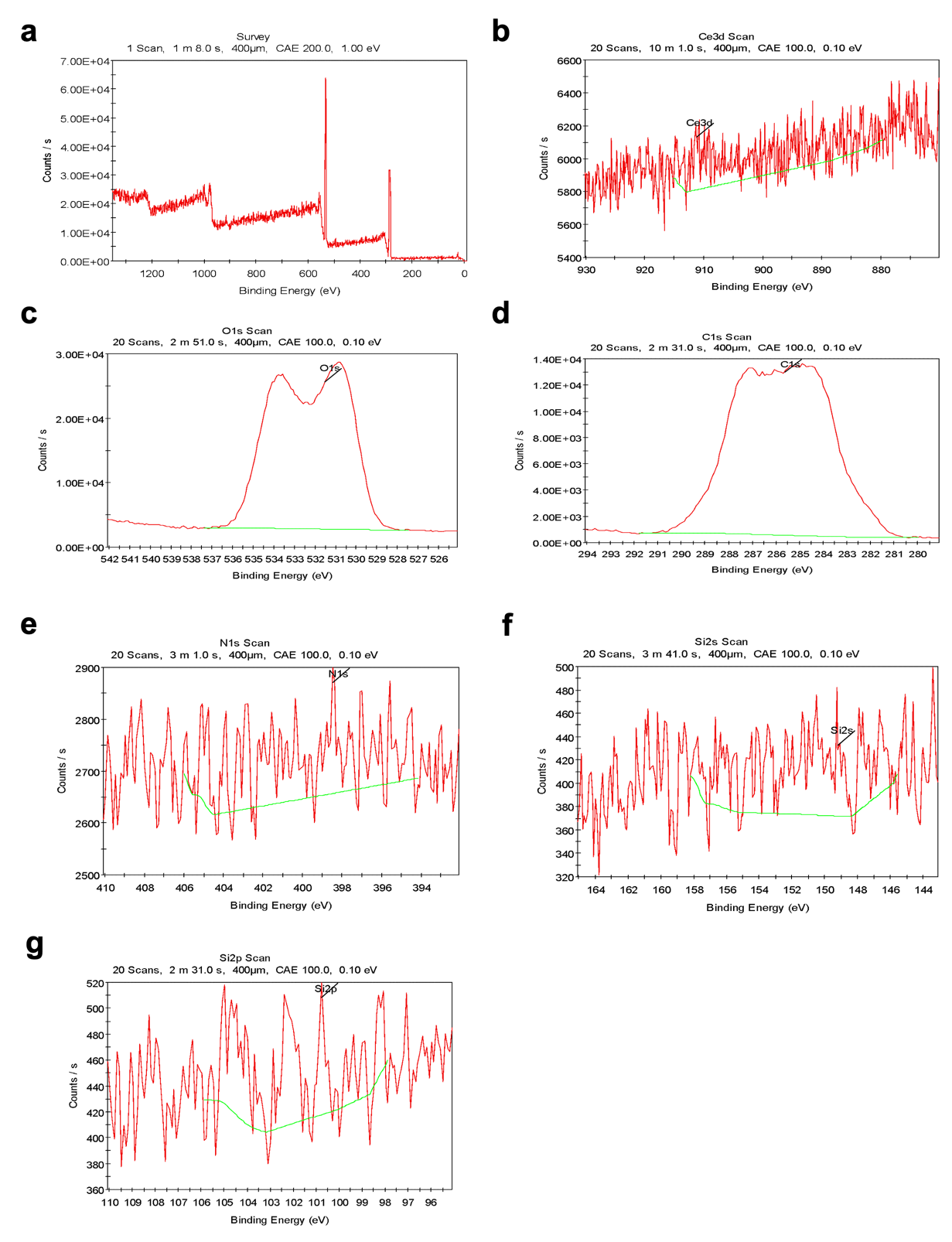


**Fig. S7.** **XPS survey spectra of bare sensing paper**. (a) XPS survey spectra of bare sensing paper made of cellulose fiber and high-resolution scans of (b) Ce3d, (c) O1s, (d) C1s, (e) N1s, (f) Si2s, and (g) Si2p.


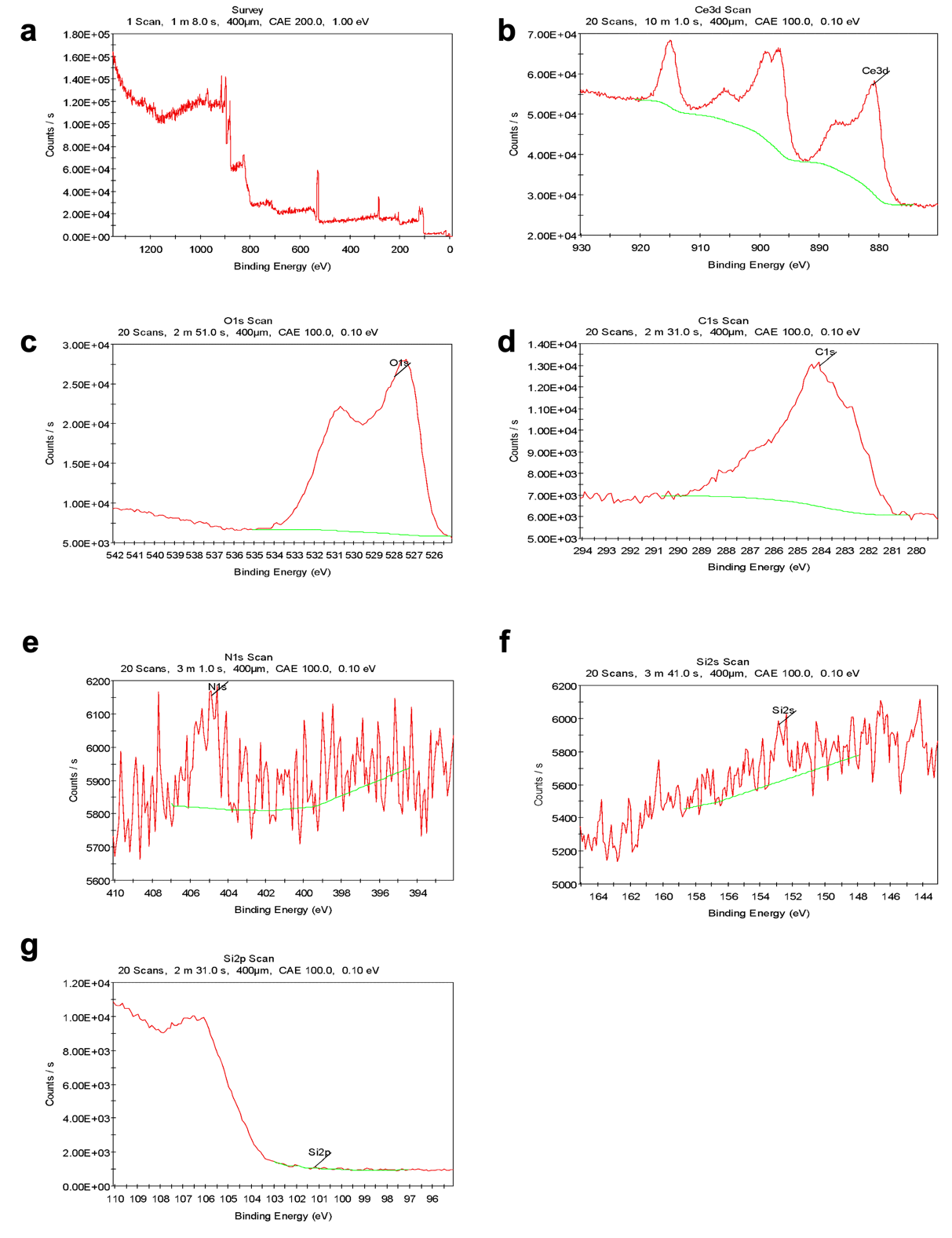


**Fig. S8. XPS survey spectra of CNPs-treated sensing paper.** (a) XPS survey spectra of CNPs-treated sensing paper and high-resolution scan of (b) Ce3d, (c) O1s, (d) C1s, (e) N1s, (f) Si2s, and (g) Si2p.


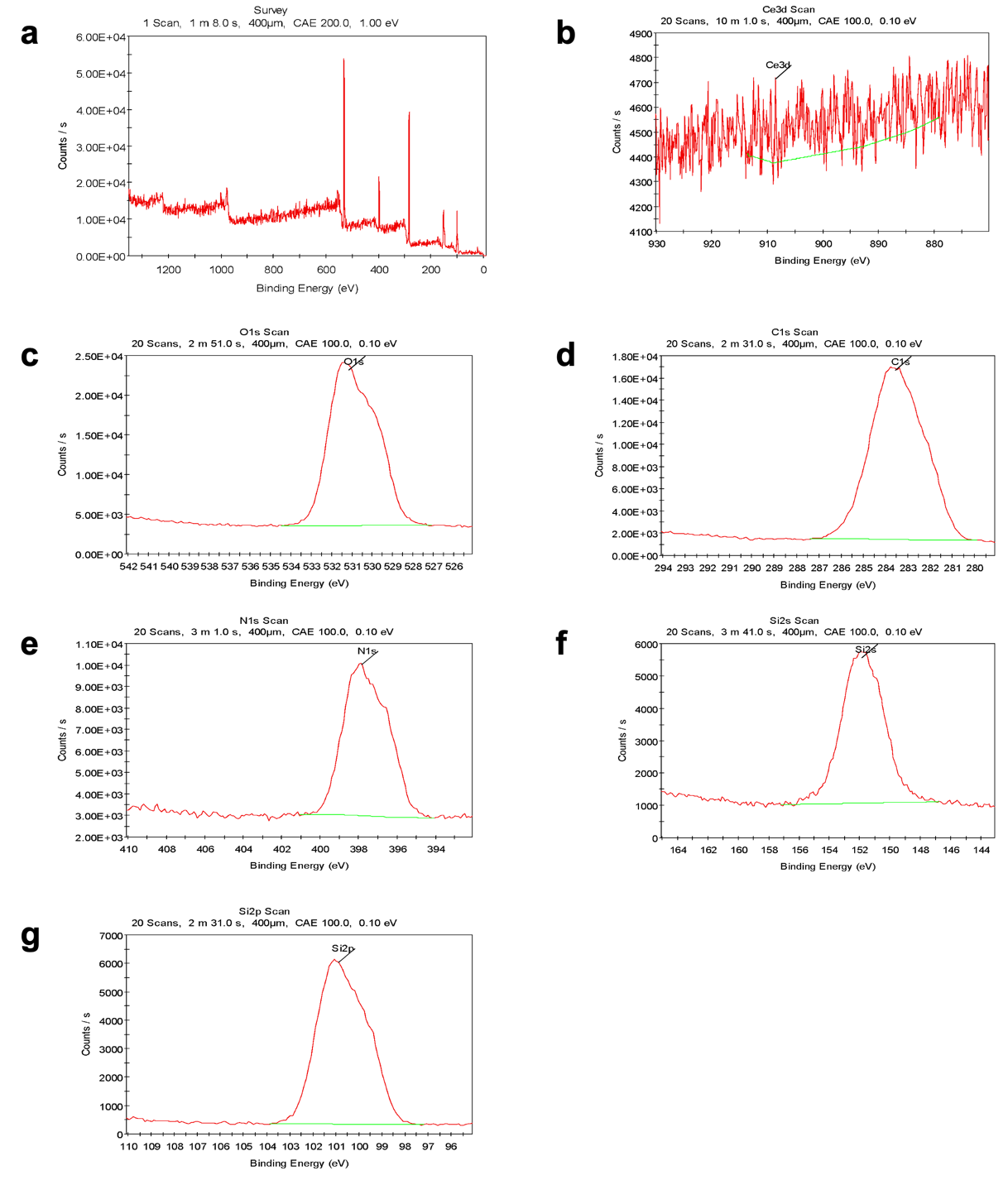


**Fig. S9. XPS survey spectra of CNPs/APTS-treated sensing paper.** (a) XPS survey spectra of CNPs/APTS-treated sensing paper and high-resolution scan of (b) Ce3d, (c) O1s, (d) C1s, (e) N1s, (f) Si2s, and (g) Si2p.


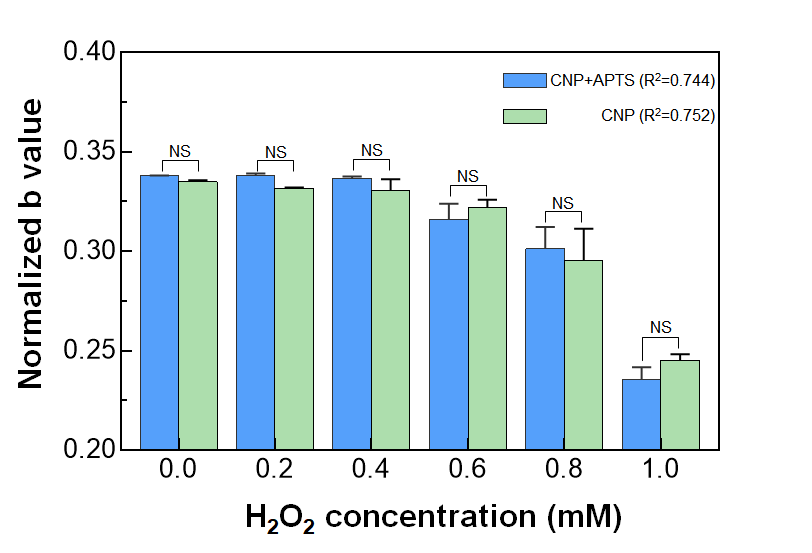


**Fig. S10. Comparison of normalized b values before and after APTS treatment in CNPs conjugation at the different concentration of H_2_O_2_.**


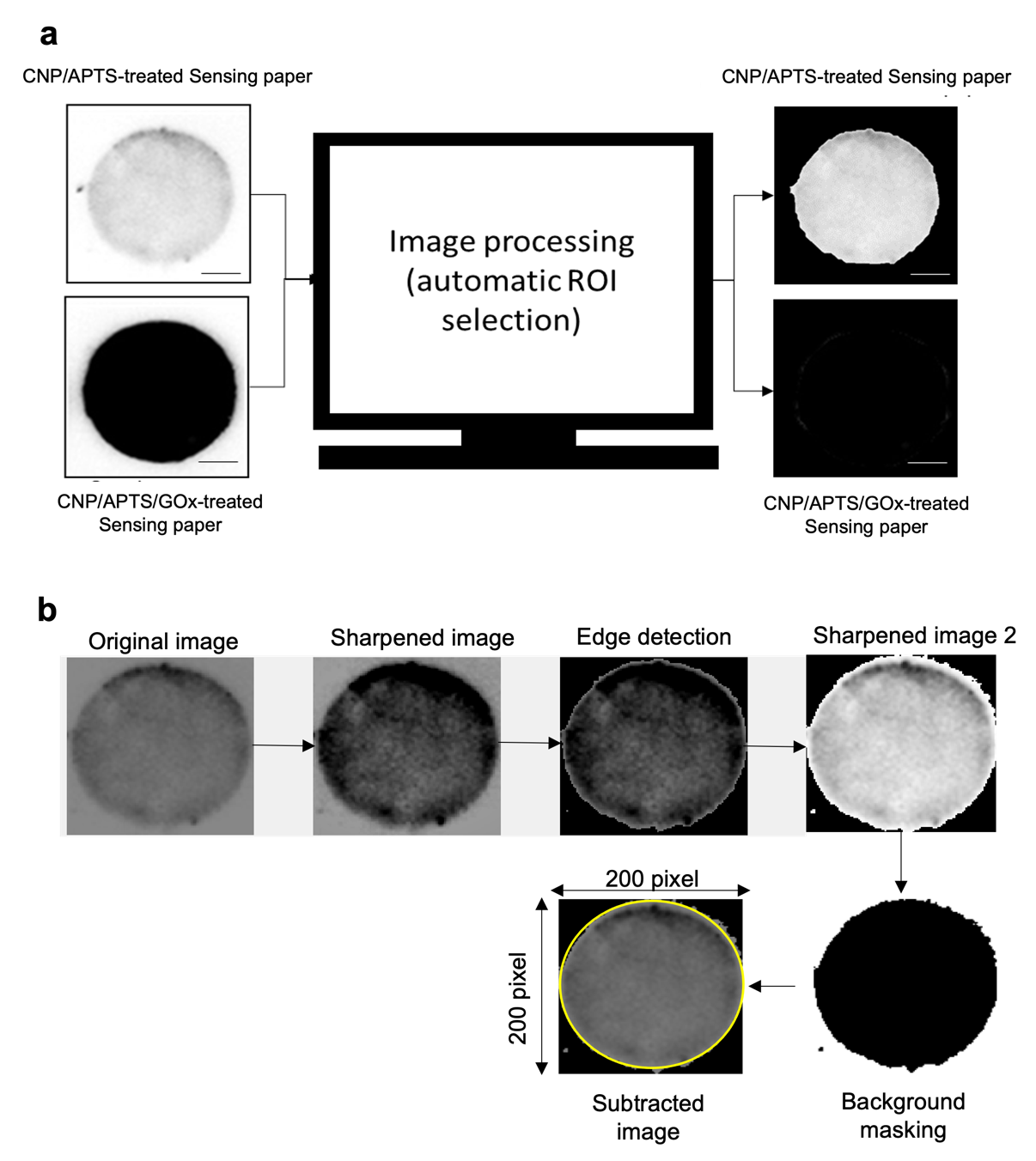


**Fig. S11.** **The overall process for calculating total intensity and uniformity with an immunofluorescence image (inverted image) of CNPs/APTS-treated or CNPs/APTS/GOx-treated sensing paper.** (a) Image processing algorithm to valuated the CNPs/APTS-treated sensing paper or CNPs/APTS/GOx-treated sensing paper. **(b)** The overall image process for calculating the GOx treatment on the CNPs/APTS-treated sensing paper.


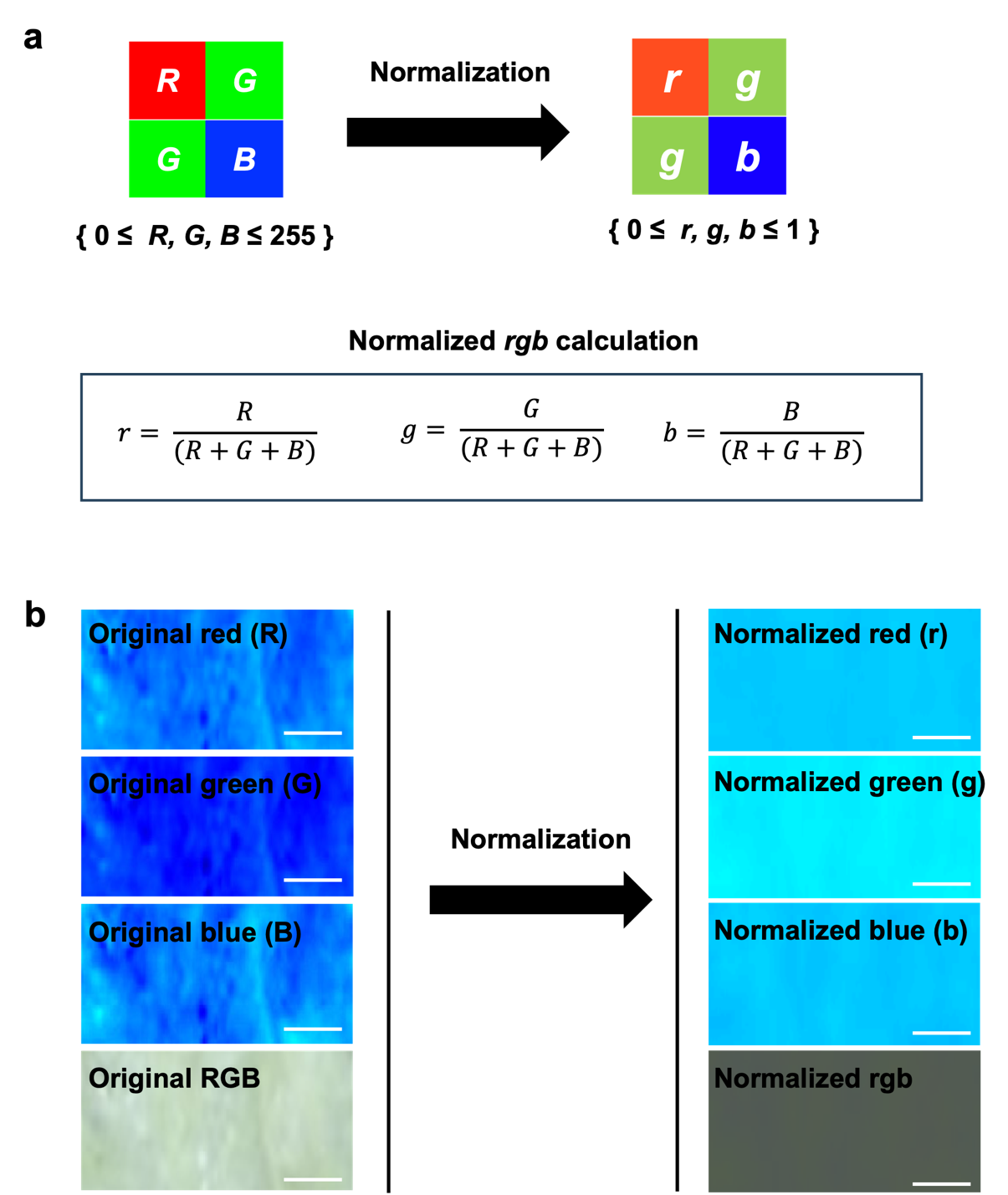


**Fig. S12. The comparison and image processing between original *RGB* and normalized *rgb* image. (a)** The normalization equation with images. **(b)** The distribution images of RGB and rgb were acquired from the original and normalized channels, respectively.


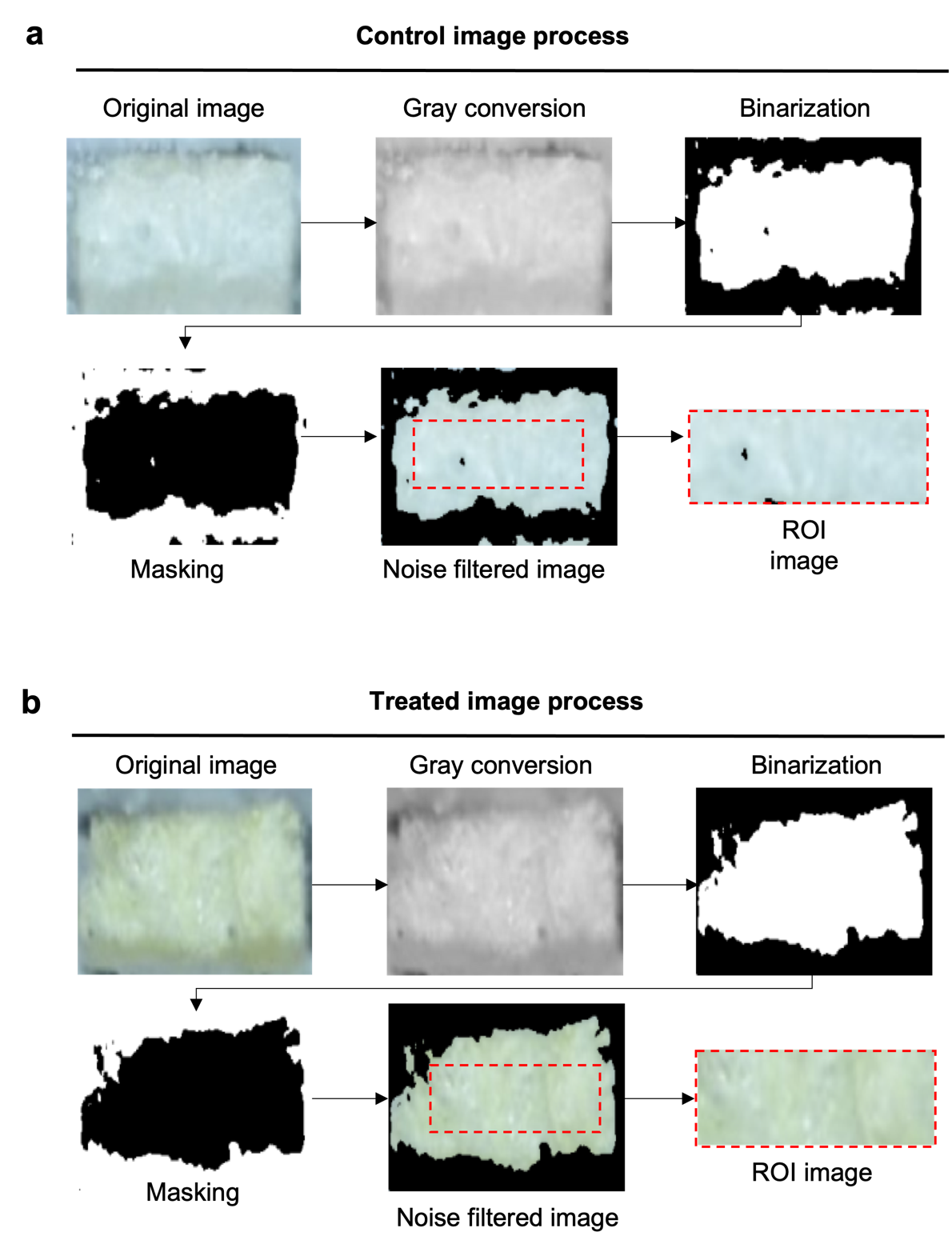


**Fig. S13.** **The overall process to estimate the color change of the sensing paper in the action chamber of the SD strip biosensor in response to tear glucose concentration**. **(a)** control image process with the original image. **(b)** normalized image process with the original image.


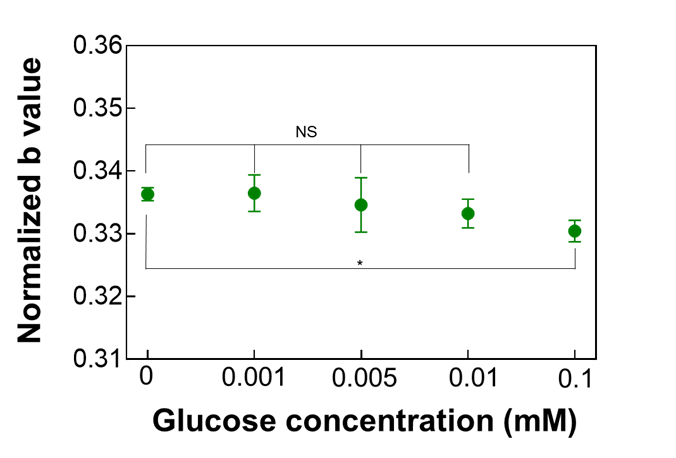


**Figure S14.** **Assessment of the Limit of Detection (LOD) for Low Glucose Concentrations.**


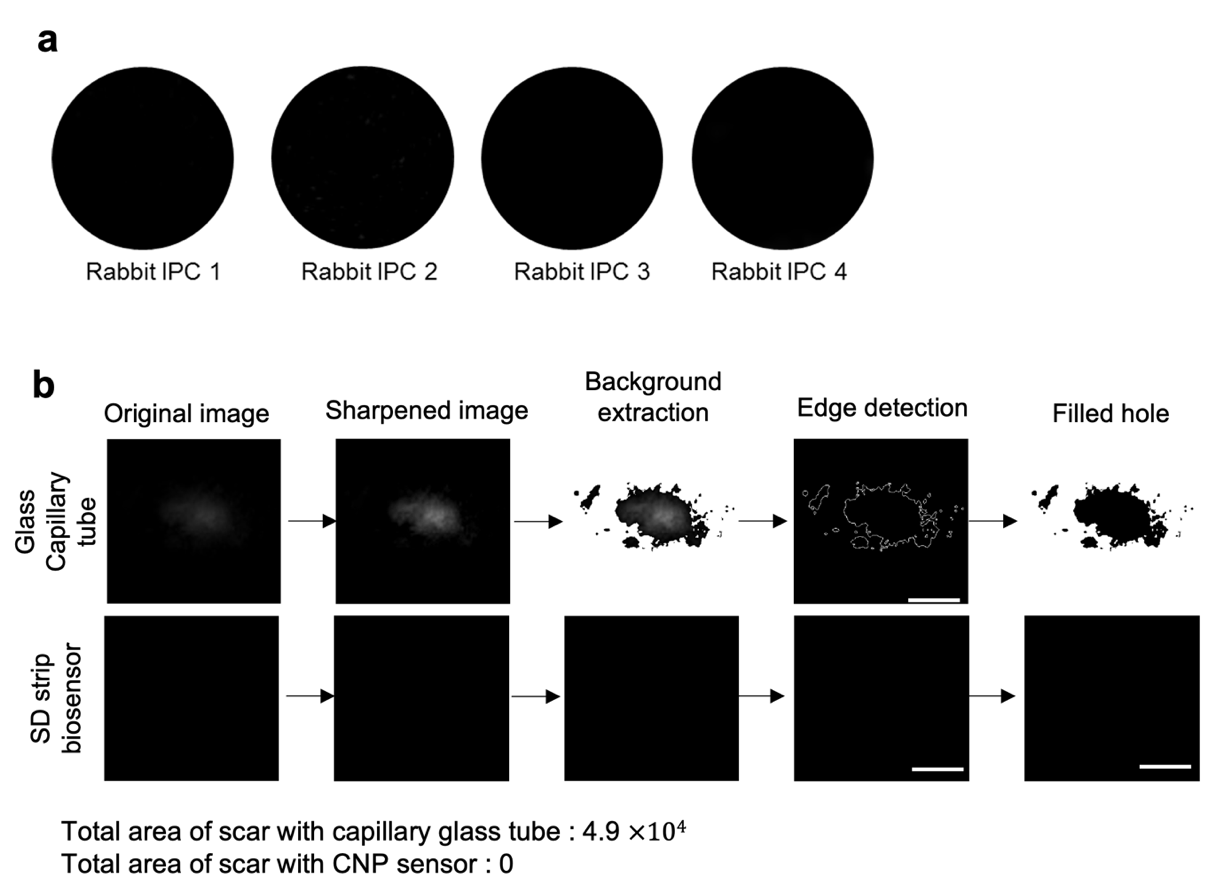


**Fig. S15.** **Image analysis of eye damage using fluorescent dye after touching the SD strip biosensor to the IPC of the rabbit eye.** **(a)** Fluorescence images of an eye after repetitive touch with the SD strip biosensor. **(b)** The overall image process of calculating the total area damaged by a glass capillary tube or the SD strip biosensor.
